# Sexual dimorphism in LC-MS/MS-based vitamin D metabolite profiling and nutrition-acquired biochemical osteomalacia among adolescents

**DOI:** 10.3389/fnut.2025.1696230

**Published:** 2025-10-09

**Authors:** Shaun Sabico, Nasser M. Al-Daghri, Amal Alenad, Yousef Al-Saleh, Malak N. K. Khattak, Kaiser Wani, Abdullah M. Alnaami, Jean-Yves Reginster, Majed S. Alokail, Etienne Cavalier

**Affiliations:** ^1^Chair for Biomarkers of Chronic Diseases, Department of Biochemistry, College of Science, King Saud University, Riyadh, Saudi Arabia; ^2^Department of Biochemistry, College of Science, King Saud University, Riyadh, Saudi Arabia; ^3^Department of Medicine, Health Oasis Hospital, Riyadh, Saudi Arabia; ^4^Protein Research Chair, Department of Biochemistry, College of Science, King Saud University, Riyadh, Saudi Arabia; ^5^Department of Clinical Chemistry, University of Liège, CIRM, CHU de Liège, Liège, Belgium

**Keywords:** vitamin D metabolites, osteomalacia, Arab adolescents, 25-hydroxyvitamin D, vitamin D metabolite ratio, VMR

## Abstract

**Introduction:**

We previously reported a high prevalence of biochemical osteomalacia among apparently healthy Arab adolescents using combined mineralization markers. This study examined whether advanced LC-MS/MS–based vitamin D metabolite profiling, including the vitamin D metabolite ratio (VMR), can serve as indicators of biochemical osteomalacia in Arab adolescents.

**Methods:**

A total of 976 age- and body mass index-matched adolescents (522 girls, mean age 14.9 ± 1.8 years, body mass index, BMI 23.0 ± 5.9; 454 boys, mean age 14.9 ± 1.7 years, BMI 23.7 ± 5.8) were included in this cross-sectional study. Anthropometrics and biochemical parameters [glucose, lipid profile, calcium (Ca), inorganic phosphorus (Pi), alkaline phosphatase (ALP)] were measured using routine assays. Circulating vitamin D metabolites [24,25(OH)₂D (24, 25 VD), VD2, VD3, total VD] were quantified using LC-MS/MS, and VMR calculated as [24,25 VD/VD] × 100. Deficiency cut-offs were: VD <30 nmol/L, 24,25 VD <3.0 nmol/L, VMR <4%. Biochemical osteomalacia was defined as ≥ 2 abnormal markers (low VD, high ALP, low Ca, or low Pi).

**Results:**

All vitamin D metabolites were significantly lower in the biochemical osteomalacia group. Overall, VD showed the highest predictive value (AUC 0.71, Youden index 0.40). Stratified analyses revealed VMR as a modest marker in girls (AUC 0.60), while VD3 performed best in boys (AUC 0.77, Youden index 0.60).

**Conclusion:**

VD metabolites as a single test are modest predictors of biochemical osteomalacia in adolescents and differ in accuracy according to sex. Findings in this study should be interpreted as exploratory rather than diagnostic, serving to generate hypotheses and lay groundwork for future clinical and public health applications.

## 1 Introduction

Osteomalacia arises from defective mineralization of the bone matrix, affecting the entire skeletal system and dentition, and in young children often coexists with rickets, a growth plate disorder with more apparent clinical and radiological features (1). It remains a significant yet under-recognized condition in adolescents, particularly in regions with very high prevalence of vitamin D deficiency, such as the Middle East and Saudi Arabia in particular (2–4). In our previous work, we identified a high prevalence of biochemical osteomalacia among apparently healthy Arab adolescents using a combination of altered mineralization markers, including low serum 25(OH)D (VD), low calcium (Ca), low inorganic phosphorus (Pi), and elevated alkaline phosphatase (ALP) (5). While serum VD is a widely used indicator of vitamin D status, it does not fully reflect the complexity of vitamin D metabolism or bone health.

Recent developments in vitamin D metabolism led to the establishment that it is not a single compound, but rather a collective term given to a group of over 50 metabolites (6), with 2 main forms extensively studied: cholecalciferol [25(OH)D_3_] (VD3) and ergocalciferol [25(OH)D_2_] (VD2) (6). This was achieved through advancements in analytical methods, particularly liquid chromatography–tandem mass spectrometry (LC–MS/MS), which simultaneously provides multiple metabolite assessment (7). Since then, several other vitamin D metabolites have been investigated, particularly 24,25-dihydroxyvitamin D [24,25(OH)₂D] (24, 25 VD), a catabolic by-product of VD, and the vitamin D metabolite ratio (VMR: 24,25(OH)₂D ÷ 25(OH)D × 100), both of which have been proposed as potentially more specific markers of disordered bone mineralization. Among athletes, for instance, VD metabolites, including free and bioavailable VD, were significantly associated with muscle strength and jump performance, highlighting their potential role in athletic performance (8). Preliminary work on a large pediatric cohort revealed that, given the same VD concentration, some children had already begun catabolizing VD and exhibited measurable circulating 24,25 VD, while others did not, suggesting that the vitamin D threshold may be individualized (9). In another large cohort (*n* = 940) of healthy young army recruits, a strong inverse association was observed between serum VD and VMR, which also significantly correlated with increasing parathyroid hormone (PTH) levels. This observation provided a mechanistic insight into the vitamin D–PTH axis, which supports not only the physiological role of 24,25(OH)₂D, but also reinforces VMR as a measure of functional vitamin D deficiency (10). In older populations, evidence from the SarcoPhAge cohort of community-dwelling older adults indicated that VMR correlates more strongly with PTH levels than either 25(OH)D or 24,25(OH)₂D alone, and that low VMR (defined as <4%) was associated with the highest all-cause mortality risk, supporting its value as a functional marker of vitamin D deficiency (11). Cumulatively, these findings suggest that VMR may offer improved clinical utility over traditional vitamin D measures in assessing deficiency and related health risks.

Despite growing evidence supporting the utility of VMR in elucidating the complex regulation of vitamin D metabolism, important knowledge gaps remain. In particular, the diagnostic accuracy of VMR and other VD metabolites for detecting biochemical osteomalacia in adolescents has not been clearly established. This is especially relevant given the unique physiological demands of growth and bone mineralization during adolescence, coupled with known sex-specific differences in vitamin D status (12), bone turnover, and susceptibility to mineralization defects (13, 14). Understanding whether VMR offers superior or complementary diagnostic value in this context could refine screening strategies, enable earlier detection, and inform targeted interventions for at-risk male and female adolescents. This study aimed to assess whether VD metabolites, including VMR, can serve as indicators of biochemical osteomalacia in a large cohort of Saudi adolescents, and to determine their predictive performance across sex-specific subgroups.

## 2 Materials and methods

### 2.1 Study participants

This cross-sectional analysis utilized clinical data from the biochemical osteomalacia database maintained by the Chair for Biomarkers of Chronic Diseases (CBCD), King Saud University (KSU), Riyadh, Saudi Arabia (5, 15). The project was conducted in partnership with the Saudi Ministry of Education and involved recruiting healthy Saudi adolescents aged 12–17 years from 60 randomly selected preparatory and secondary schools, drawn from more than 600 schools in Riyadh to ensure a representative sample of the target population. The primary aim was to determine the prevalence of biochemical osteomalacia in Saudi Arabia and explore its relationship with dietary vitamin and mineral intake. Inclusion criteria required participants to be Saudi nationals, free from acute illnesses, physically and mentally able to participate in school activities, and willing to provide informed consent/assent, fasting blood samples, and dietary recall data. Non-Saudis, those without consent, and individuals deemed medically unfit were excluded.

### 2.2 Data collection

Demographic and anthropometric data were retrieved from the database. Biochemical parameters, including fasting glucose, glycated hemoglobin (HbA1c), lipid profile (triglycerides, total and HDL-cholesterol), ALP, Pi and Ca have been previously evaluated (5, 15). All these measurements were done at the Chair for Biomarkers of Chronic Diseases (CBCD), KSU, Riyadh, Saudi Arabia, using a routinely calibrated biochemical analyzer (Konelab, Vintaa, Finland) with manufacturer-supplied quality control samples (Thermo Fisher Scientific, Espoo, Finland).

### 2.3 Vitamin D metabolites

Measurement of circulating vitamin D metabolites was carried out at the Department of Clinical Chemistry, CIRM, University of Liège, Belgium. Serum total VD, VD2, VD3 and 24,25 VD were quantified using a validated liquid chromatography–tandem mass spectrometry (LC–MS/MS) method certified by the Centres for Disease Control and Prevention (CDC) (11). The vitamin D metabolite ratio (VMR) was calculated as: VMR = [24,25 VD/VD] × 100. For this study, deficiency thresholds were defined as total VD < 50 nmol/L (16), 24,25 VD < 3.0 nmol/L, VD2 limit of quantification (LOQ) < 1.8 nmol/L, and VMR < 4%, based on established cut-offs (11). A different cut-off for low total VD was used for the operational definition of biochemical osteomalacia.

### 2.4 Biochemical osteomalacia

For this study, biochemical osteomalacia was defined as ≥2 abnormalities among four serum markers: low total VD (<30 nmol/L), elevated ALP (age- and sex-specific ranges), and/or low calcium (<2.1 mmol/L) or Pi (age- and sex-adjusted ranges) as used previously (5).

### 2.5 Data analysis

Data were analyzed using SPSS (version 22 Chicago, IL, United States). Continuous data were presented as mean ± standard deviation (SD) for Gaussian variables, and non-Gaussian variables were presented in median (25th and 75th percentiles). Categorical data were presented as frequencies (n) and percentages (%). All continuous variables were tested for normality using the Kolmogorov–Smirnov test. Non-Gaussian variables were log-transformed prior to parametric analysis. Independent Student T-Test and Mann–Whitney U test were performed to compare mean and median differences in Gaussian and Non-Gaussian variables, respectively. Logistic regression analysis was performed using biochemical osteomalacia as the dependent variable with vitamin D metabolites as independent variables to determine odds ratios (OR) with 95% confidence intervals (CI). Receiver operating characteristic (ROC) and area under the curve (AUC) analyses were performed for all VD metabolites, with the exception of VD2 (only 3 values above LOQ) to identify cut-offs for biochemical osteomalacia based on sensitivity, specificity and Younden J index. Positive predictive value (PPV) was calculated as true positive (TP)/(TP + false positive, FP), while negative predictive value (NPV) was calculated as true negative (TN)/(TN + false negative, FN) (17). A *p*-value <0.05 was considered statistically significant.

## 3 Results

### 3.1 Participant characteristics

Of the 976 Saudi adolescents (522 girls, 454 boys) included, 9.8% (*n* = 96, 83 girls and 13 boys) had biochemical osteomalacia. It is worth noting that among those with biochemical osteomalacia, girls outnumbered boys by approximately 6:1. Table 1 shows the differences between participants with and without biochemical osteomalacia. Compared to controls, the biochemical osteomalacia group had significantly higher hip circumference (*p* = 0.01) and diastolic blood pressure (*p* = 0.01). No differences were seen in age, BMI and BMI z-score. Among the biochemical parameters, the controls had significantly higher mean total cholesterol (*p* = 0.01), serum Ca (*p* < 0.001) and Pi (*p* < 0.001), with a slightly higher mean HbA1c than the biochemical osteomalacia group. No differences were seen in fasting glucose, triglycerides, HDL-cholesterol and circulating ALP. Similarly, and with the exception of VD2, all vitamin D metabolites were significantly lower in the biochemical osteomalacia group as compared to controls (all *p* < 0.001) (Table 1).

**Table 1:** General characteristics of participants with and without biochemical osteomalacia.

Parameters	Control	B. Osteomalacia	*p*-value
N (G/B)	880 (439/441)	96 (83/13)	
Age (years)	14.9 ± 1.7	14.9 ± 1.6	0.98
BMI (kg/m^2^)	23.2 ± 5.9	23.8 ± 5.8	0.35
BMI Z-score	−0.017 ± 1.0	0.084 ± 0.98	0.35
Waist (cm)	70.2 ± 18.5	72.7 ± 15.4	0.20
Hips (cm)	84.1 ± 21.7	89.9 ± 17.5	0.01
WHR	0.85 ± 0.24	0.81 ± 0.10	0.17
Systolic BP (mmHg)	117.5 ± 14.2	117.8 ± 15.6	0.86
Diastolic BP (mmHg)	71.2 ± 11.6	74.4 ± 12.7	0.01
Biochemical parameters			
Glucose (mmol/l)	5.3 ± 1.3	5.1 ± 0.5	0.23
HbA1c (%)	5.1 ± 0.9	5.0 ± 0.5	0.05
Triglycerides (mmol/l)	1.14 ± 0.6	1.08 ± 0.6	0.32
Total Cholesterol (mmol/l)	4.6 ± 0.9	4.3 ± 0.9	0.01
HDL-Cholesterol (mmol/l)	1.1 ± 0.3	1.1 ± 0.4	0.98
Calcium (mmol/l)	2.5 ± 0.4	2.2 ± 0.5	<0.001
Phosphorous (mmol/l)	1.6 ± 0.4	1.1 ± 0.34	<0.001
ALP (U/l)	63.2 ± 36.5	62.2 ± 37.8	0.81
Vitamin D metabolites			
Total VD (nmol/l)	33.1 (23.3–44.6)	24.2 (18.9–29.3)	<0.001
24, 25 VD (nmol/l)	0.88 (0.33–1.70)	0.33 (0.31–0.76)	<0.001
VD3 (nmol/l)	33.07 (23.3–44.02)	24.2 (18.9–29.3)	<0.001
VD2 (nmol/l)	2.29 ± 0.6	2.25 ± 0.0	0.59
VMR	2.73 (1.7–4.1)	1.86 (1.4–2.9)	<0.001

Data presented as N and mean ± SD and median (1st–3rd) percentiles; *p*-value significant at 0.05 and 0.01 levels.

### 3.2 Sex-stratified analysis

In Table 2, participants were stratified according to sex to determine clinical differences in girls and boys with and without biochemical osteomalacia. In girls, the biochemical osteomalacia group had significantly lower total cholesterol (*p* = 0.002), Ca and Pi (both *p* < 0.001) than controls. The same group had significantly lower total VD (*p* = 0.04), 24,25 VD (*p* = 0.002), VD3 (*p* = 0.04), and VMR (*p* = 0.004). The rest of the parameters including all anthropometrics were not significantly different between groups. In boys, the biochemical osteomalacia group also had significantly lower Ca and Pi (both *p* < 0.001) than controls, as well as significantly lower total VD (*p* < 0.001), 24,25 VD (*p* = 0.007) and VD3 (*p* < 0.001). The rest of the variables were comparable, including VMR.

**Table 2:** Differences between girls and boys with and without biochemical osteomalacia.

Parameters	Girls		*p*-value	Boys		*p*-value
	Control	B. Osteomalacia		Control	B. Osteomalacia	
	*N* = 439	*N* = 83		*N* = 441	*N* = 13	
Age (years)	14.8 ± 1.8	14.9 ± 1.7	0.77	14.9 ± 1.6	14.4 ± 1.3	0.29
BMI (kg/m^2^)	22.8 ± 5.9	23.7 ± 5.7	0.21	23.6 ± 5.8	24.5 ± 6.4	0.60
BMI Z-score	−0.09 ± 1.01	0.07 ± 0.97	0.21	0.052 ± 0.99	0.204 ± 1.09	0.60
Waist (cm)	72.1 ± 11.8	73.9 ± 12.2	0.09	68.1 ± 23.4	64.1 ± 28.6	0.56
Hips (cm)	91.0 ± 13.7	92.9 ± 12.9	0.25	76.9 ± 25.6	69.3 ± 28.8	0.32
WHR	0.80 ± 0.11	0.80 ± 0.09	0.99	0.89 ± 0.3	0.91 ± 0.09	0.89
Systolic BP (mmHg)	115.9 ± 14.2	117.7 ± 16.1	0.33	119.1 ± 14.1	118.7 ± 12.3	0.93
Diastolic BP (mmHg)	73.5 ± 11.7	75.6 ± 12.5	0.14	68.9 ± 10.9	66.3 ± 10.9	0.41
Biochemical parameters						
Glucose (mmol/l)	5.2 ± 0.9	5.2 ± 0.5	0.99	5.5 ± 1.6	5.1 ± 0.5	0.37
HbA1c (%)	5.0 ± 0.8	4.9 ± 0.5	0.22	5.3 ± 0.9	5.3 ± 0.3	0.87
Total Cholesterol (mmol/l)	4.6 ± 0.9	4.3 ± 0.9	0.002	4.5 ± 1.03	4.4 ± 0.9	0.75
HDL-Cholesterol (mmol/l)	1.1 ± 0.3	1.1 ± 0.3	0.36	1.0 ± 0.3	1.1 ± 0.4	0.67
Triglycerides (mmol/l)	1.0 ± 0.5	1.1 ± 0.5	0.89	1.2 ± 0.6	1.2 ± 0.7	0.92
Ca (mmol/l)	2.5 ± 0.4	2.2 ± 0.5	<0.001	2.6 ± 0.4	2.1 ± 0.6	<0.001
Pi (mmol/l)	1.5 ± 0.3	1.1 ± 0.3	<0.001	1.6 ± 0.4	1.2 ± 0.3	<0.001
ALP (U/l)	56.4 ± 34.5	61.7 ± 39.4	0.22	70.1 ± 37.3	65.8 ± 26.5	0.68
Vitamin D metabolites						
Total VD (nmol/l)	24.9 (19–36)	24.1(19–19.4)	0.04	39.3 (32–48)	25.9 (18–29)	<0.001
24, 25 VD (nmol/l)	0.33 (0.30–1.1)	0.33 (0.3–0.7)	0.002	1.2 (0.7–2.1)	0.33 (0.3–1.4)	0.007
VD3 (nmol/l)	24.9 (19–36)	24.1(19–29.4)	0.04	39.3 (31.9–48.2)	25.9 (18.1–29.2)	<0.001
VD2 (nmol/l)	2.3 ± 0.7	2.2 ± 0.01	0.66	2.3 ± 0.50	2.2 ± 0.001	0.81
VMR	2.3 (1.5–3.6)	1.8 (1.3–2.9)	0.004	3.1 (1.9–4.4)	2.2 (1.6–3.2)	0.053

Data presented as N and mean ± SD and Median (1st–3rd) Percentiles. *p*-value significant at 0.05 and 0.01 levels.

### 3.3 Associations and diagnostic performance of VD metabolites

Table 3 shows the ORs (95% CI) of the different VD metabolites with respect to biochemical osteomalacia from the cut-offs used. The odds of biochemical osteomalacia was significantly associated with low 24, 25 VD (<3 nmoL/L) [OR 8.0 (95% CI 1.1–58.1); *p* = 0.04], low VD3 (≤50 nmoL/L) [OR 6.0 (95% CI 1.9–19.3); *p* = 0.002], low total VD (≤50 nmoL/L) [OR 6.1 (95% CI 1.9–19.7); *p* = 0.002] and low VMR (≤4.0) [OR 3.2 (95% CI 1.6–6.2); *p* < 0.002]. All VD2 values were >1.8 and, as such, were not included in the diagnostic performance analysis.

**Table 3:** Associations of biochemical osteomalacia with LOQ and VMR.

Parameters	Cut-offs	Control	B. Osteomalacia	OR (95% CI)	*p*-value
*N*		*N* = 821	*N* = 128		
G/B		468/353	45/83		
24,25 VD (nmol/l)	≥3 nmol/l	67 (7.7)	1 (1.0)	1 8.0 (1.1–58.1)	0.04
	<3 nmoL/L	798 (92.3)	95 (99.0)		
VD3 (nmol/l)	≤50 nmol/l	724 (83.7)	93 (96.9)	6.0 (1.9–19.3) 1	0.002
	>50 nmol/l	141 (16.3)	3 (3.1)		
	LOQ > 1.8	862 (99.7)	96 (100)		
Total VD (nmol/l)	≤50 nmol/l	722 (83.5)	93 (96.9)	6.1 (1.9–19.7) 1	0.002
	>50 nmol/l	143 (16.5)	3 (3.1)		
VMR	>4.0	232 (26.8)	10 (10.4)	1 3.2 (1.6–6.2)	<0.001
	≤4.0	633 (73.2)	86 (89.6)		

Data presented as *N* (%). Odd ratio (95% CI). *p*-value is significant at the 0.05 and 0.01 levels.

### 3.4 Sensitivity and specificity of vitamin D metabolites in predicting biochemical osteomalacia

**Figure 1:**
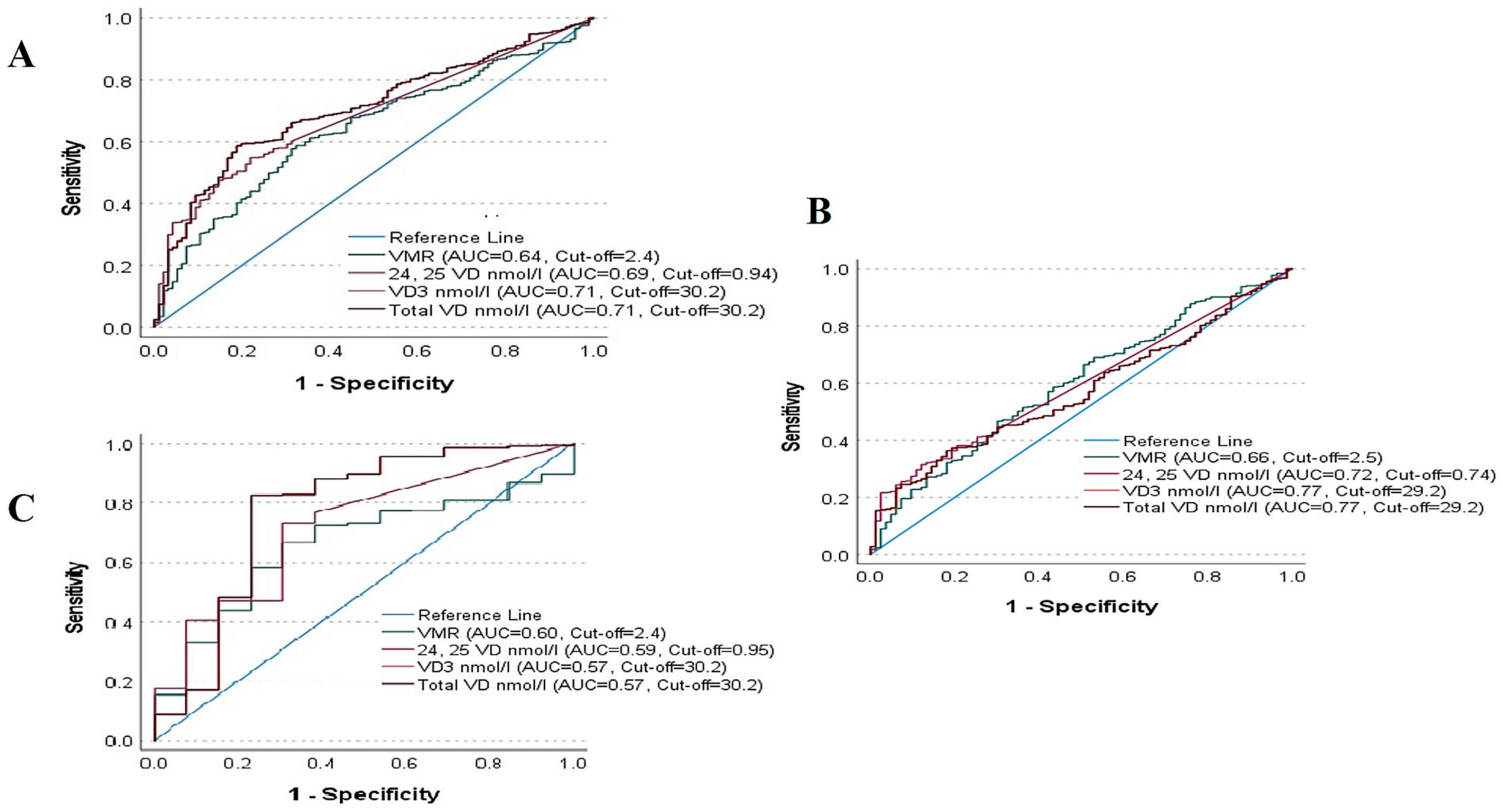
AUC Analysis of VD metabolites for biochemical osteomalacia in **(A)** all Participants, **(B)** boys and **(C)** girls.

Table 4 shows the AUC analysis of VD metabolites in predicting biochemical osteomalacia. In the overall cohort, VD (<30 nmol/L) had the highest AUC (0.71, 95% CI 0.66–0.76; *p* < 0.001) and Youden’s index (0.40), with an optimal cut-off of 30.2 nmol/L, sensitivity 59%, specificity 81%, PPV 0.11, and NPV 0.98. When stratified according to sex, in girls, VMR (≤4.0) performed comparatively strongest (AUC 0.60, 95% CI 0.53–0.66; *p* = 0.003), but still with modest sensitivity (47%) and specificity (70%). In boys, VD (<30 nmol/L) again showed the highest accuracy (AUC 0.77, 95% CI 0.60–0.94; *p* = 0.001), with sensitivity 83%, specificity 77%, and Youden’s index 0.60. AUCs were plotted in Figure 1.

**Table 4:** AUC, optimal cut-off values, sensitivity, specificity and Youden index.

Parameters	AUC (95%) CI	Cut-off	*p*-value	Sensitivity (%)	Specificity (%)	PPV	NPV	Younden Index J
Overall								
24, 25 VD (nmol/l)	0.69 (0.64–0.73)	0.94	<0.001	0.48	0.84	0.11	0.98	0.32
VD3 (nmol/l)	0.71 (0.66–0.76)	30.2	<0.001	0.59	0.81	0.11	0.97	0.40
Total VD (nmol/l)	0.71 (0.66–0.76)	30.2	<0.001	0.58	0.81	0.11	0.98	0.40
VMR	0.64 (0.58–0.69)	2.4	<0.001	0.58	0.68	0.12	0.96	0.26
Girls								
24, 25 VD (nmol/l)	0.59 (0.53–0.65)	0.95	0.002	0.32	0.88	0.17	0.96	0.20
VD3 (nmol/l)	0.57 (0.51–0.63)	30.2	0.021	0.36	0.82	0.02	0.83	0.18
Total VD (nmol/l)	0.57 (0.51–0.63)	30.2	0.020	0.36	0.81	0.17	1.00	0.17
VMR	0.60 (0.53–0.66)	2.4	0.003	0.47	0.70	0.18	0.92	0.17
Boys								
24, 25 VD (nmol/l)	0.72 (0.58–0.85)	0.74	0.002	0.73	0.70	0.03	1.00	0.43
VD3 (nmol/l)	0.77 (0.60–0.94)	29.2	0.001	0.83	0.77	0.02	0.97	0.60
Total VD (nmol/l)	0.77 (0.61–0.94)	29.2	0.001	0.83	0.69	0.04	1.00	0.52
VMR	0.66 (0.54–0.78)	2.5	0.011	0.67	0.69	0.04	0.98	0.36

Data presented AUC (95% CI), Cut-off point, sensitivity, specificity and Younden Index. *p*-value is significant at the 0.05 and 0.01 levels.

## 4 Discussion

The present study provides the most extensive sex-stratified evaluations of VD metabolites—including total VD, 24,25 VD, VD2, VD3, and VMR as potential indicators of biochemical osteomalacia among adolescents from a homogenous Arab ethnic group. Part of the novelty lies in the integration of advanced LC–MS/MS–based VD metabolite profiling with sex-stratified diagnostic performance in rarely investigated populations such as adolescents. We observed that, except for VD2, all VD metabolites were significantly lower in adolescents with biochemical osteomalacia, with total VD demonstrating the highest overall diagnostic accuracy. However, notable sex differences emerged: VMR performed comparatively best in girls, albeit with modest discriminative ability, whereas total VD and VD3 were superior in boys, with higher sensitivity and specificity. These findings highlight that the diagnostic performance of vitamin D metabolites is context- and sexually dimorphic, reflecting differences in vitamin D metabolism, bone turnover, and possibly lifestyle and hormonal factors during adolescence. Our findings align with prior evidence that VD is a robust but imperfect marker of bone health (18–20), particularly in settings with widespread VD deficiency (21–23).

The sexual dimorphism observed in VD metabolites and VMR reinforce the physiological and hormonal influences on VD metabolism during adolescence as documented in our previous investigations (24, 25). The same is true for the elderly, where sex-specific thresholds of VD status for physical function and frailty have been observed (26, 27). VD expression shows strong sexual dimorphism through its bidirectional interaction with estrogen. Estrogen decreases CYP24A1 (VD inactivation) and increases VDR expression, while VD downregulates aromatase, influencing estrogen production. This interplay is evident in immune-mediated diseases, where estrogen levels modulate VD’s immunomodulatory effects (e.g., in systemic lupus erythematosus) and where VD can counteract estrogen-driven pro-inflammatory pathways (e.g., in rheumatoid arthritis) (28). Environmental factors also play a role, such as differential sun exposure, cultural and lifestyle factors. These external aspects are more pronounced in the population used in the present study, since Saudi females are fully covered when outdoors and are less physically active than their male counterparts, making them inherently predisposed to VD deficiency (29). Given the hormonal reasons that may partially explain sexual dimorphism in VD catabolism as well as cultural factors, the rationale as to why VMR performed better in girls may still need further exploration.

The modest predictive abilities of single VD metabolites in the overall cohort suggest that relying on a single marker may be insufficient for screening biochemical osteomalacia in adolescents. The superior performance of VMR in girls supports the concept that indices reflecting both synthesis and catabolism of vitamin D could better capture subtle metabolic alterations in certain subgroups (9). This aligns with emerging evidence that VMR, as a functional biomarker, may provide additional clinical value over total VD alone, particularly where vitamin D status is influenced by sex hormones, growth demands, and differential sun exposure (30, 31). Conversely, the higher accuracy of VD3 and total VD in boys may reflect more stable metabolic processing of cholecalciferol and a stronger association between low absolute VD levels and mineralization defects in this group (14, 32).

The clinical implication of the present study is that while total VD measurement remains a practical first-line test, adding VMR in girls and focusing on VD3 in boys may improve diagnostic accuracy for biochemical osteomalacia. However, PPVs were very low across all metabolites, indicating not only limited clinical utility, but also emphasizing the need for a multi-marker approach combining vitamin D metabolites with conventional biochemical indices. Given the evidence available, the adoption of VMR as a marker of biochemical osteomalacia remains premature, and it should presently be viewed as a complementary rather than a replacement measure for total VD. Nevertheless, while our findings are consistent with earlier adult cohort studies linking low VMR to functional VD deficiency and adverse outcomes (11), the novelty of the present analysis lies in addressing a critical evidence gap by focusing on an adolescent population and using a biochemical osteomalacia definition that integrates multiple mineralization markers. The results also have public health relevance in Saudi Arabia and the Middle East in general, where a high prevalence of VD deficiency persists despite fortification programs (33–35), and where sex-specific screening strategies could enhance early detection of bone health risk.

The authors acknowledge several limitations. The cross-sectional design precludes causal inference and limits the ability to assess temporal relationships between VD metabolite changes and osteomalacia development. Factors such as dietary intake, sun exposure patterns, socioeconomic status and pubertal stage were not fully integrated into the diagnostic performance models. The low prevalence of biochemical osteomalacia in boys (*n* = 13) may have reduced statistical power to detect more minor associations in this subgroup. Furthermore, extremely low PPVs (often ≤0.18) limit clinical usefulness for screening as they impact real-world applicability (36). These low PPVs are also reflective of the low overall prevalence of osteomalacia in the cohort. Finally, the absence of bone histomorphometry or radiographic confirmation indicates that osteomalacia was inferred rather than directly verified. We therefore used the term ‘nutrition-acquired biochemical osteomalacia’ to emphasize that this represents a biochemical, rather than radiological, diagnosis based on a previously defined biochemical criterion (5). This limitation should be considered when interpreting the findings. Despite these limitations, a key strength of this study is the large, well-characterized, and sex-balanced adolescent cohort recruited from a wide range of schools, which enhances representativeness. The standardized biochemical assays, including CDC-certified LC–MS/MS for VD metabolite profiling, ensure high measurement accuracy (6). The use of a composite biochemical definition of osteomalacia improves diagnostic specificity compared to single-marker approaches. Furthermore, sex-stratified analyses allowed detection of clinically relevant differences that might otherwise be overlooked.

In conclusion, VD metabolites demonstrate sex-specific differences in their ability to predict biochemical osteomalacia among Saudi adolescents. Overall findings suggest that a one-size-fits-all approach to biochemical screening for osteomalacia in adolescents may be suboptimal, and that incorporating sex-specific thresholds or multi-marker algorithms could improve clinical utility. The authors emphasize that the investigation of VD metabolites in this study was performed as an exploratory approach to generate hypotheses and inform future research, rather than serve as a definitive diagnostic assessment. Future research should validate these findings in longitudinal cohorts to determine the temporal relationship between changes in VD metabolites and progression to clinically overt osteomalacia. Studies should also explore optimal sex-specific cut-offs and assess whether integrating VMR into current screening guidelines improves early detection rates. By defining metabolite-based associations with biochemical osteomalacia in youth, this study lays the groundwork for earlier detection strategies and informs public health initiatives targeting vitamin D deficiency in high-risk populations.

## Data Availability

The original contributions presented in the study are included in the article/supplementary material, further inquiries can be directed to the corresponding author.
